# Testing the Multiple Pathways of Residential Greenness to Pregnancy Outcomes Model in a Sample of Pregnant Women in the Metropolitan Area of Donostia-San Sebastián

**DOI:** 10.3390/ijerph17124520

**Published:** 2020-06-23

**Authors:** Asier Anabitarte, Mikel Subiza-Pérez, Jesús Ibarluzea, Kepa Azkona, Gonzalo García-Baquero, Carme Miralles-Guasch, Jon Irazusta, Kristina W. Whitworth, Guillem Vich, Aitana Lertxundi

**Affiliations:** 1 Biodonostia Health Research Institute, Group of Environmental Epidemiology and Child Development, San Sebastian 20014, Spain; m-subiza@euskadi.eus (M.S.-P.); mambien3-san@euskadi.eus (J.I.); kepa.azkona95@gmail.com (K.A.); ggbmoneo@ciberesp.es (G.G.-B.); aitana.lertxundi@ehu.eus (A.L.); 2 Department of Preventive Medicine and Public Health, Faculty of Medicine, University of the Basque Country (UPV/EHU), Leioa 48940, Spain; 3 Spanish Consortium for Research on Epidemiology and Public Health (CIBERESP), Instituto de Salud Carlos III, Madrid 28029, Spain; 4 Faculty of Psychology of the University of the Basque Country, San Sebastian 20018, Spain; 5 Health Department of the Basque Government, Sub-directorate of Public Health of Gipuzkoa, San Sebastian 20013, Spain; 6 Department of Geography, Prehistory and Archaeology, University of the Basque Country, Vitoria 01006, Spain; 7 Geography Department, Autonomous University of Barcelona, Cerdanyola del Vallès 08193, Spain; carme.miralles@uab.cat (C.M.-G.); Guillem.Vich@uab.cat (G.V.); 8 Institute of Environmental Science and Technology (ICTA), Autonomous University of Barcelona, Cerdanyola del Vallès, Barcelona 08193, Spain; 9 Department of Physiology, Faculty of Medicine, University of the Basque Country (UPV/EHU), Leioa 48940, Spain; jon.irazusta@ehu.eus; 10 Center for Precision Environmental Health, Department of Medicine, Section of Epidemiology and Population Sciences, Baylor College of Medicine, Houston, TX 77030, USA; Kristina.Whitworth@bcm.edu; 11 ISGlobal (Barcelona Institute for Global Health), Barcelona 08036, Spain

**Keywords:** mediators, maternal health, natural effects models, urban exposures, GIS

## Abstract

Residential greenness may positively impact diverse human health indicators through the reduction of air pollution, the improvement of psychological health, and the promotion of physical activity. Previous studies indicate a weak but positive association with pregnancy outcomes. Our aim was to test the multiple pathways from residential greenness to pregnancy outcomes model, using residential NO_2_ concentrations, psychological health, and moderate-to-vigorous physical activity (MVPA) during the first trimester of pregnancy, in a sample of 440 pregnant women residing in Donostia, Spain. Three metrics of residential greenness were calculated around each participant’s home address: normalized difference vegetation index (NDVI) within 300 m, and green space (>5000 m^2^) availability within 300 and 500 m. Residential NO_2_ concentrations, psychological health, and MVPA were explored as mediators of the associations between these metrics and the following pregnancy outcomes: birth weight (BW), low birth weight (LBW), prematurity, small for gestational age (SGA), and large for gestational age (LGA). Educational attainment, parity, and body mass index (BMI) were treated as covariates. Counterfactual mediation analyses showed very low to null statistical support for an association between any of the greenspace metrics and pregnancy outcomes in the full sample. Green space availability (300 m) was associated with lower BW and showed a marginal protective effect against LGA.

## 1. Introduction

Green infrastructure, which encompasses urban forests, parks, green roofs, street trees, and flowers, provides a wide array of ecosystem services that are of great interest for human health [1]. It has been proposed that greenness’s salutogenic effects may arise from three complementary pathways [2], namely the mitigation of harmful exposures, the recovery from attentional fatigue and stress, and the encouragement of physical activity (PA) and social interactions. Current evidence supports the benefits of greenness (measured as availability of green spaces and/or normalized difference vegetation index (NDVI) near the residence), including, among others, improving cardiovascular (CV) health and reducing CV-related mortality [3], reducing obesity rates [4], increasing physical and mental health [5,6], and reinforcing social cohesion [7]. Besides, international urban planning strategies and policies point at the value green areas and infrastructures in the achievements of healthier cities [8].

Researchers are also aware of the impact of greenness on pregnancy outcomes [9], which are of utmost importance due to their association with cognitive development, medical conditions, and morbidity and mortality in later stages of life [10,11,12]. The literature in this area suggests that residential greenness weakly but significantly reduces the risk of small for gestational age and preterm birth and increases birth weight [13,14,15,16,17,18,19]. Evidence of the possible influence of green spaces on large for gestational age is scarce. To our knowledge, there is only one previous study of this question, in which support for this association was not found [20]. In a large exposome study on birth weight [21], residential greenness measured via NDVI within 100, 300, and 500 m from the mother’s residence was positively associated with birth weight and was protective against term low birth weight. However, evidence is equivocal and most of the studies cited above report significant effects on some but not all the considered pregnancy outcomes (i.e., birth weight, low birth weight, prematurity, and small for gestational age). In addition, some studies have shown negative effects of residential greenness on pregnancy outcomes [22,23].

**Figure 1:**
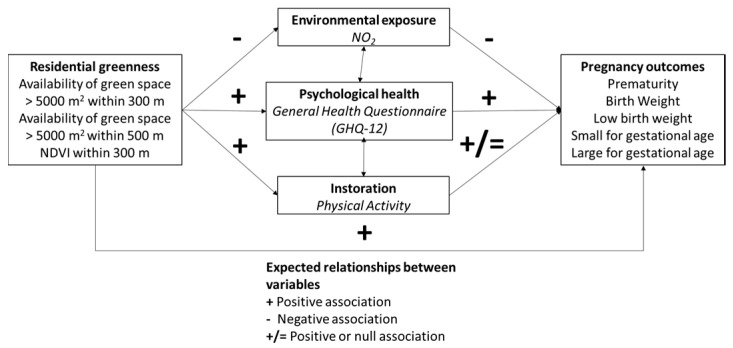
Proposed multiple pathways of residential greenness to pregnancy outcomes (MPRGPO) model and associations linking residential greenness to pregnancy outcomes in the study sample.

Kihal-Talantikite et al. [9] suggested that residential green space might have a positive effect on newborns’ health through the improvement of maternal physical and psychological health and the reduction of exposure to contaminants such as air pollution. The main objective of our study was to test the model presented in Figure 1, which is an adaptation of the one developed by Markevych et al. [2], focused on the possible effects of residential greenness on pregnancy outcomes (i.e., prematurity, birth weight, low birth weight, small for gestational age, and large for gestational age). We expect to observe a positive effect of greenness on these outcomes and expect that they will be mediated through three different pathways: (1) the reduction of exposure to air pollution, (2) the improvement of psychological health, and (3) the promotion of PA. Finally, it is also possible that residential greenness may have a positive influence on outcome variables through other pathways not considered in this study.

### 1.1. Previous Evidence Supporting the Multiple Pathways of Residential Greenness to Pregnancy Outcomes (MPRGPO) Model

We briefly review current literature on (1) the association of residential greenness and the three proposed mediators and (2) the association between those mediators and the pregnancy outcomes selected for this study (Figure 1).

First, residential greenness may mitigate air pollution. For example, vegetation removes atmospheric particles by dry deposition onto their surfaces and absorbing gaseous pollutants through their stomata [24]. In Strasbourg (France), it was estimated that urban trees absorb 7% of PM_10_ emissions in the city [25], and, according to Nowak [26], tree canopy is responsible of the absorption of 8% of NO_2_ emissions. Cai, Zhuang, and Ren [27] emphasize the ability of well-designed and interconnected green spaces in the reduction of PM_2.5_ and NO_2_ air levels, although the potential of green spaces to mitigate ozone concentrations is much more limited. Residential green space and green elements may also foster improved psychological health through the recovery from cognitive fatigue and emotional distress [28,29], and evidence points to the positive influence of residential greenness on psychological health [5,19,30]. Hence, if residential greenness enhances psychological health during pregnancy, it may subsequently have a positive effect on pregnancy outcomes (please see Epigraph 1.2.2). Indeed, in a study analyzing data from over 7000 singleton pregnancies, researchers found that residential greenness (in the form of NDVI) and a lower distance to green spaces exerted a protective role against depressive symptoms during pregnancy [31]. Finally, residential greenness (using both metrics of NDVI and availability of greenspace within 300 m of the home) has been positively associated with self-reported MVPA in European adults [30]. This effect is also apparent in other studies of green space availability and PA [32] and is likely due to the fact that green spaces promote PA by providing room for activities (e.g., walking, running, playing sports, etc.) that might not be easily performed in other settings [33].

The current literature also provides rich evidence of the connection between the proposed mediators (Figure 1) and pregnancy outcomes. Numerous studies, including systematic reviews and meta-analyses, support associations between multiple air pollutants (e.g., NO_2_, PM_2.5_, and PM_10_) and pregnancy outcomes, including those related to alterations in birth weight and gestational age [34,35]. Additionally, given remarkable changes in the biological, behavioral, and social spheres that occur during pregnancy, there has been increasing interest in studying psychological health in pregnancy. For instance, between 7–20% of pregnant women may have depressive symptoms [36,37,38]. Some studies indicate a more delicate psychological state during pregnancy than in other moments of life [39], although there is also evidence that psychological health does not vary—in statistical terms—between the pre-pregnancy or postpartum periods, or between pregnant and non-pregnant women [40]. Regardless, psychological health during pregnancy may be an important determinant of pregnancy outcomes. In a meta-analysis of eight cohort studies, Lima et al. [41] reported that high maternal stress during pregnancy increased the odds of low birth weight but not preterm delivery.

Finally, while it is well established that PA promotes physical and psychological health [42,43] for the general population, until recently, pregnant women have been advised to limit their PA [44]. However, a review by Schlüssel and colleagues [44] concluded that, in fact, PA during pregnancy reduces the risk for pre-eclampsia and gestational diabetes and failed to detect consistent adverse effects on miscarriage, low birth weight, or cesarean deliveries. More recently, a meta-analysis of randomized clinical trial and cohort studies did not detect any negative association between exercise during pregnancy and pregnancy outcomes [45]. A similar picture was obtained in two other meta-analyses [46,47]. In a study with greater than 97,000 participants, low PA patterns were associated with higher odds of preterm birth and cesarean delivery, with no effect detected for highly active women [48]. 

### 1.2. Blue Spaces and Health: Similarities to Green Spaces

The literature about the effects of greenness on human health is wide and well-consolidated. However, less attention has been paid to the salutogenic potential of blue spaces (e.g., rivers, sea, lakes, and other superficial water bodies), which have been frequently included in the “green space” category [49]. Apparently, some of the positive effects detected for greenness appear also in relation to blue spaces. Some studies have found positive associations between exposure and use of blue spaces and both general physical and psychological health [50,51,52] and increased PA [51,53]. Nevertheless, the question of whether exposure to blue spaces is positively associated with pregnancy outcomes has not been addressed to date [22].

The objective of this study was to test the Multiple Pathways of Greenness to Pregnancy Outcomes Model by analyzing the direct association of residential greenness with pregnancy outcomes and the indirect associations via the proposed mediators. Moreover, we aimed to test this model with the availability of walkable green and blue spaces, to see whether the effects of blue exposure could be comparable.

## 2. Materials and Methods 

### 2.1. Study Sample and Procedure

**Figure 2:**
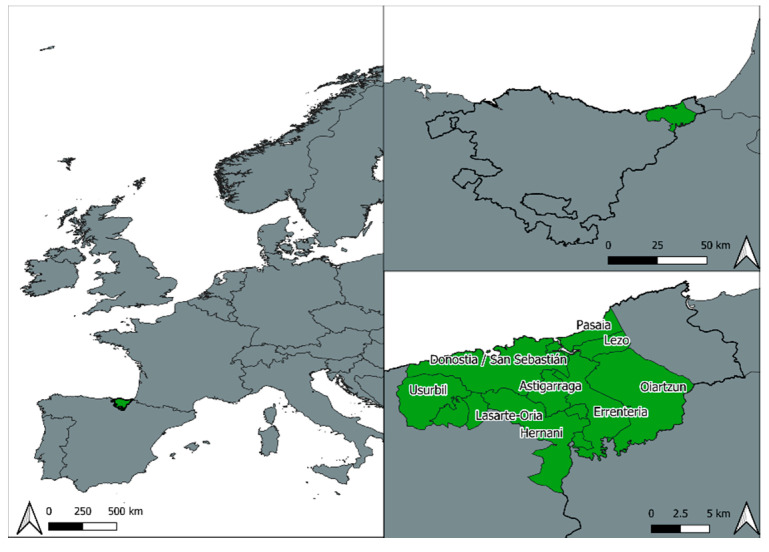
Location map of the study area.

We recruited 441 pregnant women (mean age 33.52; SD = 4.88) living in the metropolitan area of Donostia-San Sebastián (Spain). This area, located in the Northeastern region of the Basque Country, is composed by the municipalities of Astigarraga, Donostia-San Sebastián, Errenteria, Hernani, Lasarte-Oria, Lezo, Oiartzun, and Pasaia y Usurbil (Figure 2). All of these municipalities compose the functional area of the main city of the region (Donostia-San Sebastián) and maintain a semi-continuous urban scene.

Participants were recruited from among all women who attended the gynecological health service for the 12th week echography that is routinely conducted in the Basque Health Service (Osakidetza). Women residing in the study area, being able to adequately communicate in Basque or Spanish, and not having been identified with a high-risk pregnancy, were invited to take part in the study. If interested, they were led to a private room, where a researcher provided them with further information about the study, including an explanation of the implications of taking part in the study and administered informed consent. After consenting, participants were administered the study questionnaire and provided with an accelerometer (ActiGraph GT3X-BT; ActiGraph LLC, Pensacola, FL, USA). They were instructed to wear the accelerometer for one week, starting on the day of recruitment, and given information on how to return the device after its use. By the 20th week of pregnancy, and before attending the second echography, participants were contacted again and invited to wear the accelerometer for one week more. They were also given, in person, a short questionnaire. Birth information was obtained via the medical birth records in Osakidetza. The study protocol, part of the Urban Green Activity Reproductive Effect (UGARE) research project was approved by the Research Ethics Committee of the Health Department of the Basque Government (Ethical Approval Number: PI2018108). 

### 2.2. Study Instruments and Variables

#### 2.2.1. Residential Greenness

We calculated three residential greenness metrics (availability of green space with area > 5000 m^2^ within 300 or 500 m of the woman’s home and NDVI within 300 m of the home) for each of the study participants, using the geocoded of each participant’s home at the time of her recruitment. NDVI (Equation (1)) is a residential greenness metric commonly used in previous studies that indicates the level of greenness of a given area and which is calculated through the combination of the near infrared (NIR) and the red band based on satellite imagery with a 30 × 30 m resolution in the maximum vegetation period (03.08.2019) [54]. The value of the NDVI ranges from −1 to +1, with 1 being the maximum level of greenness [55]. As mentioned, we separately calculated residential NDVI within 300 and 500 m buffers of each participant’s home.

(1) $$NDVI=\frac{\left(NIR-Red\right)}{\left(NIR+Red\right)}$$

Availability of residential greenness was operationalized, using a dichotomous indicator of the presence (or absence) of a green space of area >5000 m^2^ within a 300 or 500 m buffer of the residence and accessibility by the estimation of the minimum distance (in a straight line) with a green space of the former dimensions. For both green space availability variables, we used a local layer obtained from GeoEuskadi, the spatial data service of the Basque Country.

Availability of walkable green space (of area >5000 m^2^) and blue space (the sea and main rivers) within 300 m of the residence was also calculated for the subsample of participants (*n* = 256) residing in the city of Donostia-San Sebastián. The term “walkable” means, here, those green and blue settings that are accessible and often used by citizens to walk and run (among other activities). The inclusion of spaces in the walkable category was collectively defined by three of the authors’ (AA, MS-P, and JI), who research and live in the city. This variable was only created for women who live in the municipality, as a result of the limited knowledge of the authors about the use patterns of use of green and blue spaces in the rest of the study area.

#### 2.2.2. NO_2_ Exposure Assessment

We assessed air pollution exposures by using estimates of residential NO_2_ concentration from land-use regression (LUR) models previously developed [56], which accounted for the spatial variability expected among participants. The variables included in the LUR model were (1) road length in 1000 m buffer, (2) main road length in 25 m buffers, and (3) area of low residential density in 5000 m buffers. In contrast to the original authors, we obtained the road network from the Basque Country’s IDE (Spatial Data Infrastructure in its Spanish acronym) and residential density from the CORINE 2018 Program (Coordination and Information on the Environmental Programme; CLC 2018 accessed in https://land.copernicus.eu/pan-european/corine-land-cover/clc2018), initiated by the European Commission. Once participants were assigned a LUR-based NO_2_ exposure level, we applied a time correction to account for seasonal changes. To do so, we gathered daily air-quality data from eight air-quality stations from the Basque Government Air Quality Network’s stations in the study area. Each participant was assigned to the station closest to her residence. Individual LUR values were divided by the average value of all the stations during the study period (October 2018–February 2020) and then multiplied by the daily value of the corresponding station. Hence, an individual daily value adjusted for spatial and time variation was obtained for each participant. Finally, we calculated individual average value for the whole pregnancy by compiling the exposure scores meeting the pregnancy dates.

#### 2.2.3. Physical Activity

Objective PA was determined based on the accelerometer (Actigraph wGT3X-BT set at 30 Hz) worn by the participants for two separate one-week periods during pregnancy (once in the first trimester and once during the second trimester). Participants’ PA data were used in the analyses if they had worn the device a minimum of three days of at least 10 h of use per day in each sampling period. Sleeping hours (23:00–06:00) were not taken into account, and Freedson 1987’s thresholds were used to calculate the minutes of light, moderate, and vigorous activity. This allowed us to estimate the number of minutes of sedentary behavior, as well. For the analyses, we built a composite measure reflecting MVPA by adding registered daily minutes of moderate and vigorous physical activity. Self-reported PA was also assessed through a single question in which participants were asked to define themselves as sedentary, scarcely active, moderately active, quite active, or very active [57,58] in each of the sampling periods.

#### 2.2.4. Psychological Health

Participants’ psychological health status during the first trimester was measured with the Spanish version of the General Health Questionnaire [59]. This scale comprises 12 items reflecting diverse psychological symptoms and daily functioning issues, using a 0–4 scale in which the respondent is asked to indicate whether she is experiencing them and to what degree. The sum of the answers provides a score ranging from 0–36, with higher scores indicating worse psychological states or higher amounts of stress. The internal consistency of the scale for this study was good (α = 0.75).

#### 2.2.5. Pregnancy Outcomes 

The outcome variables defined for this study were birth weight, preterm birth, low birth weight (LBW), small for gestational age (SGA), and large for gestational age (LGA). We defined preterm birth as birth <37 completed weeks of gestational age and low birth weight as <2500 g. SGA and LGA were defined as sex-specific birthweight less than the 10th percentile (SGA) or greater than the 90th percentile (SGA) for gestational age, based on the distribution of birthweights for 19,000 births in Gipuzkoa, during the period 2013–2015.

#### 2.2.6. Covariates

We also obtained a list of adjustment variables. Sex of the newborn, participant’s parity, and season of the birth were obtained from medical records. Socioeconomic status (SES, in the form of a district privation index developed for the MEDEA project-http://www.proyectomedea.org/-) was assigned to each participant, based on their residential district. Finally, body mass index (BMI) before and during pregnancy, based on the WHO classification, was calculated from self-reported data [60].

### 2.3. Data Analysis

The events of being born small for gestational age (SGA) or large for gestational age (LGA) give rise to two of the five newborn traits used as response variables in the subsequent mediation analysis. We classified newborns in the present dataset as either SGA, LGA, or normo-type. For this purpose, we first employed a second dataset, obtained from 9682 boys and 9485 girls born in Gipuzkoa during 2013–2015, to compute the 10th and 90th percentiles (i.e., 0.1th and 0.9th quantiles) for the sample distributions of newborn weights (g) at each of the gestational weeks, 25–42 (Supplementary Table S1). To estimate these quantiles, the median-unbiased estimator (<sample quantiles type 8> in Reference [61]) was used, as implemented in the R function quantile() of R software v. 3.6.1 (R Foundation for Statistical Computing, Vienna, Austria) [62]. This estimator not only is defined independently of underlying probabilistic distributions, but also Hyndman and Fan [61] found that it possesses most of the required properties of (sample) quantile estimators. Once computed by using the abovementioned second dataset, we then used the said quantile estimates to classify newborns in the present dataset as SGA or LGA if the observed weight was below the 10% threshold or above the 90% threshold, respectively; otherwise, they were considered as normo-type (i.e., neither SGA nor LGA).

**Figure 3:**
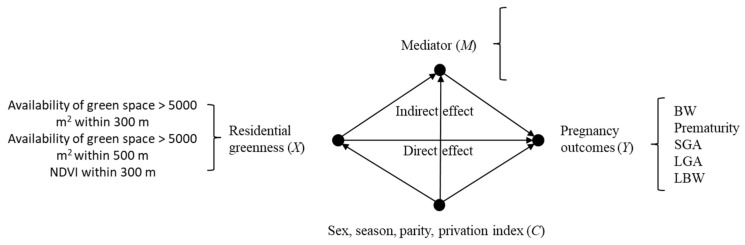
Directed Acyclic Graph representing the hypotheses tested in this research work for the whole study population. These mediation hypotheses concern the questions whether each of three variables experienced during pregnancy, namely GHQ, MVPA, and NO_2_ exposure, can be considered as intermediate mechanisms (M) through which neighborhood greenness (X) exerts its influence on each of the five newborn traits (Y), namely birth weight, prematurity, small for gestational age (SGA), large for gestational age (LGA), and low birth weight (LBW), once that it has been conditioned on the covariates (C), namely sex of the newborn, season in which the neonate was born, maternal parity, and family privation index.

In order to address the study’s objective, we used data from the whole study population to apply mediation analysis [63]. For these analyses, we used objective MVPA during the first trimester of pregnancy instead of the self-reported estimation (for accuracy reasons) or MVPA during the second trimester, as only a half of the sample participated in the second data-collection period. First, we analyzed associations between each of the primary study variables (exposures, mediators, and outcomes) with the adequate statistical procedures for each pair of comparison (Yule’s phi coefficient, Welch’s F, and chi-squared test). Mediation analysis allowed us to explore whether effects of residential greenness on the pregnancy outcomes under study (i.e., birth weight, LBW, preterm birth, SGA, and LGA) are mediated by maternal psychological health, MVPA during pregnancy, or residential NO_2_ exposure, once that we condition them on a priori covariates (i.e., sex of the newborn, season in which the neonate was born, maternal parity, and family privation index). In each pathway, we decomposed total effects into natural (also called pure) direct and indirect effects [64] via the mediation formula [65] as implemented in the R package medflex [62,66]. Total effects were thus decomposed within the framework of counterfactual outcomes [64], into natural direct and indirect effects via the mediation formula [65]. The advantage of this approach is that it allows the extension of mediation analysis to include a large class of models [67,68] that encompasses both numerical and categorical responses and exposures, as well as numerical and categorical mediators. More explicitly, we fitted imputation-based [69] natural effect models [70] to test mediation hypotheses concerning the general question whether either maternal psychological health, maternal physical activity, or residential NO_2_ exposures experienced during pregnancy act as mediators (M) of the effect of exposure (X) to neighborhood greenness (Figure 3) on any of the five newborn traits (Y) (i.e., birth weight, prematurity, SGA, LGA, and LBW), conditioned on the covariates (C) (i.e., sex of the newborn, season of birth, maternal parity, and family privation index). The sampling distributions of model parameters were approximated by using nonparametric bootstrap with 1000 replications, and these sampling distributions were then used for statistical inference (hypothesis testing). 

We also computed population-average effects, together with 95% C.I., for the natural-effects models reported (Supplementary Material Table S5 and Supplementary Material Table S6).

Additionally, as mentioned, we also used data from the subsample of the study population who resided in the municipality of Donostia-San Sebastián, to assess, using the same statistical technique, whether the same variables (i.e., psychological health, MVPA, or NO_2_ exposure) mediate the effect of either exposure to walkable blue space availability (within 300 m), walkable green space availability (within 300 m), or the union of both exposures, on each of the aforementioned five newborn traits, once we take into consideration the said covariates (see Supplementary Materials Figure S1).

In the first case (Figure 3), since there are three potential mediators (psychological health, MVPA, and NO_2_), five response variables (birth weight, prematurity, SGA, LGA, and LBW), three exposures (availability of green space >5000 m^2^ within 300 and 500 m and NDVI within 300 m), and only one set of covariates, there are sixty (4 × 5 × 3) hypotheses to be tested. In the second case (Supplementary Figure S1), there also are sixty additional hypotheses. A description of the variables involved in this research is given in Table 1.

## 3. Results

### 3.1. Testing the Multiple Pathways of Residential Greenness to Pregnancy Outcomes Model in the Study Sample

Participants were 33.52 years old on average (SD = 4.48). A total of 50.44% of the participants had no currently living children previous to the ongoing pregnancy, 36.87% had one previous child, and the rest of the sample had two or more other children. A 66.67% of the women which participated in the study were normal-weighted, 16.22% were overweight, and 6.78% were obese, according to the BMI classification system. Around two-thirds of the sample had completed tertiary education (64.60%) and were working at the time of data collection (79.94%). The sociodemographic profile of the study participants and further data on their characteristics can be found elsewhere [71].

Around three-quarters and nine-tenths of the participants lived within 300 m and 500 m of a green space with area >5000 m^2^, respectively (Table 1). NDVI scores (mean = 0.21; SD = 0.09) converge with what might be expected in urban built settings with grassland sections, street trees, and other usual elements of urban greenery. The study of mediator variables reveals that participants experienced low-to-moderate levels of psychological stress and exercised, on average, above the international PA recommendations. With regard to NO_2_, average concentration values in the study area (mean = 46.7 µg/m^3^; SD = 22.36) were higher than the average annual value in the Basque Country (mean = 22–30µg/m^3^) [71]. In relation to outcome variables, 9.3, 14.6, 4.8, and 3.3% of the births were categorized as SGA, LGA, LBW, or premature, respectively. The average birth weight was 3350 g, with a standard deviation of 500 g.

Our initial analyses did not reveal statistically significant associations between the three exposure variables and most of the mediators or outcomes (data not shown). The exceptions were a statistically significant differences in average BW by green space availability within 300 m of the residence (F = 4.91, *p* < 0.05; not found at the 500 m level) and in average residential NO_2_ concentrations by green space availability within 500 m of the residence (F = 7.20, *p* < 0.01), and a negative correlation between NDVI within 300 m and MVPA minutes (rho = −0.179, *p* < 0.001). These results indicate that participants living in the vicinity of a green space of more than 5000 m^2^ delivered children with lower average birthweights and were exposed to lower NO_2_ concentrations, and those living in greener environments performed less activity at moderate-to-vigorous intensities per day. We also found that participants who delivered LBW children were less active than the rest of the sample (F = 7.20; *p* < 0.05).

The main results of the natural effects mediation models are shown in Table 2. Overall, the data did not confirm the existence of either a direct link between residential greenness with the pregnancy outcomes selected for this study or an indirect association between the former and the latter through the three proposed mediators. The only statistically significant effect we observed was a negative direct effect of green space availability in 300 m on BW, which was observed in the model using NO_2_ as mediator. This means that participants living close to green spaces delivered children weighing, on average, 140 gless at birth than participants living further from green spaces. The direct effect in the models using the other two mediators was only marginal (*p* < 0.10). Another marginal negative direct effect of green space availability in 300 m was detected for LGA when GHQ was used as a mediator. More extensive information about Table 2 can be seen in Supplementary Material Table S2.

### 3.2. Testing the Pathways from Residential Greenness or Blueness to Pregnancy Outcomes in a Study’s Subsample

As reported in Section 2.3., we also included an indicator of exposure to blue spaces (availability of a walkable blue space within a 300 m radius of the residence), to check whether it showed significant direct or indirect effects on the study outcomes. Of the 256 participants included in this secondary analysis, only 77 (30.1%) lived within 300 m of a walkable blue space. Separately, none of the models using green and blue space availability (Supplementary Material Table S3) showed statistically relevant direct or indirect effects on the outcomes, with the sole exception of a marginally significant increase of 36 g (*p* = 0.066) in BW in children delivered by mothers with green-space availability in 300 m in the model using NO_2_ concentrations as mediator (Supplementary Material Table S4). We also built the corresponding mediation models, in order to test the combined effects of having a walkable green space or blue space, and the condition was met by less than half of the sample (107, 41.8%). None of the coefficients, which are shown in Table 3, reached statistical significance. More extensive information about Table 3 can be seen in Supplementary Material Table S4.

## 4. Discussion

This study tested the Multiple Pathways of Greenness to Pregnancy Outcomes model (adapted from [2,9]). According to this model, and congruently with recent research (e.g., [15,21]), we expected that residing near green spaces to (1) reduce exposure to ambient NO_2_, (2) strengthen psychological health, and (3) promote PA, all of which would exert positive direct and/or indirect effects on a set of pregnancy outcomes in a sample of 441 pregnant women in the metropolitan area of Donostia-San Sebastián. However, the results of our analyses provided little support for this model. 

None of our three metrics of residential greenness (i.e., availability of green space of area >5000 m^2^ within 300 or 500 m of the residence, and NDVI within a 300 m radius of the residence) showed consistent or significant associations with the mediators and outcomes included in our model. Our data showed some relevant links (e.g., green space availability in 300 m with NO_2_ concentrations and lower PA patterns in participants delivering LBW children), but, on the whole, they do not support the MPGRH model. Indeed, only a single direct effect coefficient was found to be statistically significant (at an alpha level of 0.05), indicating that participants living within 300 m of a green space delivered children with birthweights, on average, 140 g less than their counterparts, a result opposite of the evidence gathered by James et al. [54] that showed a positive association between residential greenness (NDVI) and birth weight.

Finally, we conducted a secondary analysis among the subsample of participants living in the city of Donostia-San Sebastián, including availability of blue spaces as additional exposure variable. In these mediation models, we also added a measure of walkable green or blue space, meaning that the spaces should be frequently used by the citizenship. However, the results of this secondary analysis were largely unchanged from the main analyses (apart from a marginally significant effect of greenness on birth weight).

### 4.1. Interpretation of Results in Context of Available Evidence

First of all, it should be taken into account that, even though the theoretical assumptions of the relationship between residential greenness and pregnancy outcomes are solid (e.g., greenness reduces air pollution, and this, in turn, reduces prematurity), even the studies with supporting evidence do not show a clear and consistent pattern in all the exposure types and pregnancy outcomes examined. In our study, and contrary to previous evidence [13,14,15,16,17,18,19,21,54], green space availability was negatively and significantly associated with birth weight. The only previous study (to our knowledge) to examine greenness with LGA [20] did not find an association, though we did find that green space availability marginally reduced the odds of LGA in our sample. The fact that we have not found any statistically significant direct connection between NDVI and pregnancy outcomes might lie, partially, on the NDVI values. Studies showing such links had sample average NDVI levels greater than ours—0.5 ± 0.1 in 300 m and 0.546 (0.089) (Median (IQR)) in 500 m [21,23], which double the ones in this study (0.21 ± 0.1). Similarly, results from other pregnancy cohorts also report NDVI values higher than these in the 300 m radius (0.42 ± 0.1 and 0.51 ± 0.1) [72]. This means that our sample not only lived in less green areas than samples in other studies, but the variability between participants’ scores is also low. There is at least one study reporting a protective effect of greenness on pregnancy outcomes with lower NDVI values [13]; however, it is plausible that those effects may appear only above a certain NDVI threshold. This is, that residential environments might not only need to have some greenness but need to be green enough to strengthen the pregnancy outcomes of their inhabitants. For instance, negative associations between residential NDVI and MVPA have been observed, and that may happen because large green spaces might be placed in locations with lower building densities and mixed uses. Hence, residents in those areas may have lower access to services and destinations by foot and then resort to less active means of transport. Finally, our sample was also very homogenous in terms of the green space availability metrics; 75% of participants lived within 300 m of a >5000 m^2^ green space, and 88% in 500 m, which might have also limited our ability to find statistically significant effects.

Regarding our findings dealing with indirect effects, NO_2_ concentrations assigned to participants in our study are higher than in other studies [21,23,73]. This might be due to the use of another source of information to determine the roads and create the LUR model in our study. Beelen’s [56] LUR model was calculated by using a dataset that was no longer available at the time we had to calculate NO_2_ exposure levels. Thus, we calculated road-related variables by using local information layers. The average assigned NO2 value (46.70 ± 22.36) in our study strongly differs from the annual average NO2 value reported by Basque Government authorities in 2018 [74]. In addition, LUR models developed in the European Study of Cohorts for Air Pollution Effects (ESCAPE) project are reliable in terms of detecting inter-individual variability, but they might overestimate NO_2_ concentration values, which may also affect our estimations, even though the use of the Government’s air quality network values to estimate temporal variability may have partially reduced overestimation. The effects of greenness on pregnancy outcomes are expected to be protective, whereas air pollution is negatively associated with pregnancy outcomes. 

In the analyses with the subsample of residents in the main city of the study area, we saw a marginal effect of walkable green space availability in 300 m on birth weight. In comparison to the full sample analyses, we only included green spaces that are frequently used by the city residents, due to the relevance of not only the actual availability of green spaces but their user-oriented design and their use, as well [75]. Even so, we believe the inclusion of walkable green space in the model might provide a more accurate characterization of the effects of greenness on pregnancy outcomes.

### 4.2. Strengths and Limitations

There are several strengths to this study. First, MVPA was objectively measured by using an accelerometer as opposed to self-reports, which correlate only moderately to weakly with objective measures [76,77]. Indeed, we have previously shown, using data from this same research project [71], that objectively measured and self-reported MVPA during the first trimester of pregnancy were only moderately correlated (*r* = 0.44). The reasons for the limited association between both measurement modalities are described in that work. Secondly, we have used three metrics of exposure to residential greenness: availability of a green space of size >5000 m^2^ within 300–500 m of women’s residence and NDVI within a 300 m radius of women’s residence. We have also advanced a possible way of increasing the validity of the measures in availability by considering only the spaces that are frequently used by citizens. An important limitation of Geographic information system (GIS)-based greenness analyses is that participants can live objectively near a park, garden, or green lot but may not fully benefit from it due to use restrictions (e.g., private property and insecurity). This lack of relevant contextual information has been identified as one of the flaws of GIS-based methodologies by Gidlow et al. [78], and this solution might contain its possible deleterious effects. 

Given we found no evidence against the null hypothesis that psychological health, air pollution, and physical activity experienced during pregnancy do not mediate the effect of neighborhood greenness on birth weight, prematurity, SGA, LGA, and LBW, readers might ask whether our results arise from pervasive error in our hypothesis testing, i.e., whether systematic type II error occurred. Likewise, readers might question our results by suggesting that these results might have arisen from model misspecification [79] in our counterfactual-based mediation analysis. 

The issue of model misspecification has been dealt with elsewhere, so here we only discuss the possibility that systematic type II error occurred in our data analyses. Type II error occurs when one does not reject a null hypothesis when this hypothesis is in fact false. In general terms, for a given statistical technique and a fixed probability of type I error, α (which, in our case, was set at the traditional level of 0.05), the probability of type II error, β (or, complementarily, the statistical power, 1 − β) when testing for an effect of a certain size depends on sample size [80]. Thus, the first of the hypothetical objections that a reader might pose boils down to ask whether we used samples of sufficient size. To evaluate whether our study results might arise from insufficient sample size, we used results from theoretical simulations, as follows. Loeys et al. [81] used sample sizes ranging from *n* = 25 to just *n* = 200, to estimate statistical power under a variety of simulated mediation analysis scenarios. They found that, under unfavorable scenarios, power for detecting the most-difficult-to-detect cases of indirect effects could be as low as approximately 0.45% when sample size was as large as *n* = 200, though (still under unfavorable scenarios) power was in most cases substantially higher (between 70–90%). By contrast, power for detecting the most-difficult-to-detect cases of indirect effects under scenarios now more favorable was at least c.70% when sample size was *n* = 200, though, under those favorable scenarios, power was in most cases substantially higher (between 95–99%). For those reasons, given that most of our cases fall not within the category of the most-difficult-to-detect cases (since the prevalence of most outcomes is greater than circa 5%), and also because our analyses used samples of substantially higher sizes (*n* = 256 in the case of the analysis limited to Donostia-San Sebastian; *n* = 436 in the case of the more general analysis), we believe it unlikely that our results arise from pervasive type II error (except in the case of the outcome prematurity, which had a prevalence of about just 5%). 

Besides, in a simulation study comparing both the relative RMSE (root-mean-square error) and the relative bias of imputation-based [79] natural effect models [70] fitted with the R package medflex [66], which are the kinds of models used in the research here presented, to RMSE and bias obtained by means of other approaches fitted via several other packages and software systems (such as the R package mediation SAS macros), Lange and Starkopf [82] found that, for sample sizes comprising 250–500 subjects, imputation-based natural effect models achieved minimum levels of both relative bias (which, in fact, was nearly null) and relative RMSE (between 0.5–0.25).

Nevertheless, this study is also affected by several limitations. We are not aware of the distribution of NDVI scores and green and blue space availability for the Basque population, and therefore we cannot estimate whether our sample is representative in those terms. Besides, according to registered MVPA levels [78], we can conclude that our sample is very active. The inclusion of both PM (2.5 and 10) and noise would have strengthened our study due to the known associations between those and pregnancy outcomes [16,83,84,85,86,87,88]. However, NO_2_ is correlated with other specific air pollutants (e.g., particulate matter) and is often used as a marker of traffic-related air pollution and noise pollution [16,89], and it was the only available indicator of air pollution, so this limitation is relatively controlled. According to official data, 6.89% of children delivered in the Basque Country are LBW and a 6.36% are delivered preterm [90]. Our data show lower proportions of LBW and preterm infants (4.8 and 3.3%, respectively), and this might be indicative of a self-selection bias and might suggest that our results are not generalizable to the target population (only 33.77% of the contacted women decided to take part in the study). Finally, another limitation that may have affected our study is that participants might have performed PA in places far from their residence [91]. If so, urban greenness could have had a positive effect on pregnancy outcomes which we have been unable to detect. This issue could be solved by using data from the Global Navigation Satellite System (GNSS).

Future studies on the multiple pathways of residential greenness to pregnancy outcomes could overcome these limitations by imputing to participants more adjusted NO_2_ values or even better measuring direct personal exposure with ad hoc devices (as done in Reference [92]). Widening the set of air pollution variables would also help to determine greenness contribution to its containment and subsequent potential positive effects on pregnancy outcomes. Finally, identifying which are the most-used green settings might assist for the correct weighting of the effects of interest here. Apart from our initiative for the analyses with the subsample (relying on authors’ knowledge), surveys or interviews might be key to get this information. In this line, the use of GNSS devices might enlighten this line of research by providing us with real information about the use of green spaces in residential contexts. 

## 5. Conclusions

We could not find support to the hypotheses underlying the Multiple Pathways of Residential Greenness to Pregnancy Outcomes model, as our data failed to show significant direct or mediated associations between diverse measures of both residential greenness and pregnancy outcomes in the whole study sample. Analyses with a section of the sample, and using an improved GIS-based determination of green space availability, showed a promising trend effect of the former in birth weight through NO_2_ concentration levels. Nevertheless, and in view of the results reported by other researchers in the area, it is greatly needed to keep exploring the role of greenness in pregnancy outcomes and possible mediators involved.

## Figures and Tables

**Table 1:** Description and distribution of study variables ranged by role in the analyses.

Role in the Analyses	Variable	Type	*n*	*n* Missing	Condition Is Met	Minimum	Maximum	Mean	SD	Median	Q1	Q2	IQR
Exposure	Green space availability within 300 m	Binary	435	1	325 (74.7%)	N/A	N/A	N/A	N/A	N/A	N/A	N/A	N/A
	Green space availability within 500 m	Binary	435	1	384 (88.3%)	N/A	N/A	N/A	N/A	N/A	N/A	N/A	N/A
	Neighborhood greenness (NDVI) within 300 m	Quantitative continuous	435	1	N/A	0.07	0.5	0.21	0.09	0.2	0.14	0.26	0.11
Mediator	Mental health	Quantitative discrete	373	63	N/A	0	29	10.8	4.07	10	8	13	5
	Average NO_2_ during the whole pregnancy	Quantitative continuous	400	36	N/A	11.3	226	46.7	22.36	40.2	33	56	23
	MVPA during the first trimester	Quantitative continuous	338	98	N/A	4	124	39.9	21.65	36.3	24.1	53.4	29.35
Response	Birth Weight	Quantitative continuous	400	36	N/A	1600	4900	3350	488.62	3340	3060	3640	580
	Prematurity	Binary	398	38	13 (3.3%)	N/A	N/A	N/A	N/A	N/A	N/A	N/A	N/A
	SGA (small for gestational age)	Binary	397	39	37 (9.3%)	N/A	N/A	N/A	N/A	N/A	N/A	N/A	N/A
	LGA (large for gestational age)	Binary	397	39	58 (14.6%)	N/A	N/A	N/A	N/A	N/A	N/A	N/A	N/A
	LBW (low birth weight)	Binary	400	36	19 (4.8%)	N/A	N/A	N/A	N/A	N/A	N/A	N/A	N/A
Covariate	Parity	Quantitative discrete	400	36	N/A	0.00	10.00	0.71	0.89	1.00	0.00	1.00	1.00
	Privation Index	Quantitative discrete	435	1	N/A	1.00	5.00	2.42	1.28	2.00	1.00	3.00	2.00
					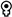	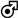	Spring	Summer	Autumn	Winter	N/A	N/A	N/A
	Sex	Binary	401	35	196 (48.9%)	205 (51.1%)	N/A	N/A	N/A	N/A	N/A	N/A	N/A
	Season	Nominal	398	38	N/A	N/A	82 (20.6%)	135 (33.9%)	127 (31.9%)	54 (13.6%)	N/A	N/A	N/A

Note: SD—standard deviation; Q1—quartile 1; Q2—quartile 2; IQR—interquartile range; 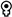
—Female; 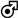
—Male.

**Table 2:** Direct and indirect natural effect coefficients and SE by residential greenness metric, mediator, and outcome.

Exposure	Prematurity			SGA			LGA			LBW			BW		
	NO_2_	GHQ	MVPA	NO_2_	GHQ	MVPA	NO_2_	GHQ	MVPA	NO_2_	GHQ	MVPA	NO_2_	GHQ	MVPA
Green availability 300 m															
Direct effect	−0.1 (2.69)	−0.71 (3.10)	−0.32 (2.61)	0.68 (0.55)	0.46 (0.56)	0.42 (0.79)	−0.52 (0.32)	−0.64 ^†^ (0.38)	−0.48 (0.37)	0.39 (2.35)	−0.20 (2.48)	0.06 (2.31)	−138.65 * (60.35)	−107.87 ^†^ (65.39)	−110.63 ^†^ (66.90)
Indirect effect	−0.17 (0.15)	0.06 (0.23)	0.01 (0.17)	−0.02 (0.06)	<0.01 (0.03)	<0.01 (0.04)	0.03 (0.04)	0.01 (0.03)	−0.012 (0.04)	−0.12 (0.11)	0.05 (0.08)	−0.016 (0.12)	6.62 (5.26)	−1.24 (4.54)	−2.06 (6.23)
Green availability 500 m															
Direct effect	0.99 (8.27)	0.02 (8.49)	0.27 (8.28)	1.12 (5.10)	1.01 (5.19)	0.95 (4.89)	−0.11 (0.54)	−0.20 (1.59)	−0.25 (0.77)	0.46 (5.76)	−0.26 (5.80)	−0.10 (6.03)	−137.35† (81.96)	−123.87 (86.26)	−133.9 (86.98)
Indirect effect	−0.50 (0.38)	0.11 (0.19)	0.01 (0.26)	−0.05 (0.14)	<0.01 (0.04)	<0.01 (0.07)	0.05 (0.08)	0.03 (0.05)	−0.02 (0.07)	−0.31 (0.25)	0.04 (0.09)	−0.01 (0.15)	15.39 (12.95)	−2.44 (5.43)	−3.97 (9.20)
															
NDVI 300 m															
Direct effect	−0.34 (0.38)	−0.5 (0.70)	−0.14 (0.49)	0.14 (0.18)	0.01 (0.19)	0.12 (0.20)	−0.05 (0.15)	0.02 (0.18)	−0.03 (0.17)	−0.07 (0.20)	−0.11 (0.25)	−0.12 (0.25)	−6.15 (22.28)	4.26 (23.12)	−4.75 (23.88)
Indirect effect	0.19 (0.14)	0.01 (0.07)	0.02 (0.08)	>−0.001 (0.02)	<0.01 (0.01)	<0.01 (0.05)	<0.01 (0.02)	<0.01 (0.01)	−0.01 (0.03)	0.08 (0.09)	<0.01 (0.03)	0.08 (0.06)	0.42 (0.60)	0.85 (2.14)	−3.46 (4.73)

Note: Coefficients are beta coefficients per units increase of each of the residential greenness metrics and the standardized mediators. SE—standard error; †—*p* < 0.10; *—*p* < 0.05; NO_2_—individual NO2 residential concentrations; GHQ—General Health Questionnaire; MVPA—moderate-to-vigorous physical activity; SGA—small for gestational age; LGA—large for gestational age; LBW—low birth weight (<2500 g); BW—birth weight.

**Table 3:** Direct and indirect natural effect coefficients and SE of green/blue availability in 300 m by mediator and outcome.

	Prematurity			SGA			LGA			LBW			BW		
	NO_2_	GHQ	MVPA	NO_2_	GHQ	MVPA	NO_2_	GHQ	MVPA	NO_2_	GHQ	MVPA	NO_2_	GHQ	MVPA
Green/blue availability 300 m															
Direct effect	−0.28 (5.10)	−0.48 (9.06)	−0.72 (6.42)	0.12 (1.48)	0.12 (0.89)	0.39 (1.71)	−0.20 (0.45)	−0.01 (0.54)	−0.35 (0.50)	−1.34 (6.81)	−1.58 (7.26)	−1.45 (6.52)	51.47 (68.73)	107.19 (72.73)	80.41 (81.38)
Indirect effect	−0.07 (2.70)	0.04 (9.77)	−0.01 (1.93)	−0.03 (0.11)	<0.01 (0.07)	−0.05 (0.13)	−0.02 (0.07)	>−0.001 (0.06)	0.06 (0.10)	−0.14 (0.45)	0.01 (1.22)	−0.10 (0.83)	21.81 (14.60)	−3.02 (12.05)	9.62 (13.91)

Note: Coefficients are beta coefficients per units increase of each of the residential greenness metrics and the standardized mediators. SE—standard error; NO_2_—individual NO_2_ residential concentrations; GHQ—General Health Questionnaire; MVPA—moderate-to-vigorous physical activity; SGA—small for gestational age; LGA—large for gestational age; LBW—low birth weight (<2500 g); BW—birth weight.

## Supplementary Materials

The following are available online at https://www.mdpi.com/1660-4601/17/12/4520/s1. Table S1: Descriptive statistics, together with 10th and 90th percentiles, for the sample distributions of newborn weights (g) at gestational weeks 25–42 in Gipuzkoa. Table S2: Direct and indirect natural effect coefficients and SE by exposure, mediator, and outcome. Table S3: Direct and indirect natural effect coefficients and SE of Green/Blue availability in 300 m by mediator and outcome. Table S4: Direct and indirect natural effect coefficients and SE by exposure, mediator, and outcome for the study subsample (*n* = 256). Table S5: Population-average effects for the natural effects models reported in Table 2, applicable to a typical individual in the population. Table S6: Population-average effects for the natural effects models reported in Table 3, applicable to a typical individual in the population. Figure S1: Directed Acyclic Graph representing the hypotheses tested in this research work for only a fraction (San Sebastián/Donostia) of the whole study population. The model is similar to that used for the whole population, except that the exposures (X) now studied are blue space availability within 300 m, green space availability within 300 m, and the union (but not the intersection) of the two former exposures (i.e. whether either a blue area, or a green area, or both, is available for the mother within 300 m around her home).
